# Investigating the DNA methylation profile of e-cigarette use

**DOI:** 10.1186/s13148-021-01174-7

**Published:** 2021-09-28

**Authors:** Rebecca C. Richmond, Carlos Sillero-Rejon, Jasmine N. Khouja, Claire Prince, Alexander Board, Gemma Sharp, Matthew Suderman, Caroline L. Relton, Marcus Munafò, Suzanne H. Gage

**Affiliations:** 1 MRC Integrative Epidemiology Unit at the University of Bristol, Bristol, UK; 2 Population Health Sciences, Bristol Medical School, University of Bristol, Oakfield House, Oakfield Grove, Bristol, BS8 2BN UK; 3 National Institute for Health Research Applied Research Collaboration West (NIHR ARC West), University Hospitals Bristol NHS Foundation Trust, Bristol, UK; 4 School of Psychological Science, University of Bristol, Bristol, UK; 5 Department of Experimental Psychology, Medical Sciences Division, University of Oxford, Oxford, UK; 6 Department of Psychological Sciences, University of Liverpool, Liverpool, UK

**Keywords:** e-cigarettes, DNA methylation, Smoking, SEE-Cigs, ALSPAC

## Abstract

**Background:**

Little evidence exists on the health effects of e-cigarette use. DNA methylation may serve as a biomarker for exposure and could be predictive of future health risk. We aimed to investigate the DNA methylation profile of e-cigarette use.

**Results:**

Among 117 smokers, 117 non-smokers and 116 non-smoking vapers, we evaluated associations between e-cigarette use and epigenome-wide methylation from saliva. DNA methylation at 7 cytosine-phosphate-guanine sites (CpGs) was associated with e-cigarette use at *p* < 1 × 10^–5^ and none at *p* < 5.91 × 10^–8^. 13 CpGs were associated with smoking at *p* < 1 × 10^–5^ and one at *p* < 5.91 × 10^–8^. CpGs associated with e-cigarette use were largely distinct from those associated with smoking. There was strong enrichment of known smoking-related CpGs in the smokers but not the vapers. We also tested associations between e-cigarette use and methylation scores known to predict smoking and biological ageing. Methylation scores for smoking and biological ageing were similar between vapers and non-smokers. Higher levels of all smoking scores and a biological ageing score (GrimAge) were observed in smokers. A methylation score for e-cigarette use showed poor prediction internally (AUC 0.55, 0.41–0.69) and externally (AUC 0.57, 0.36–0.74) compared with a smoking score (AUCs 0.80) and was less able to discriminate lung squamous cell carcinoma from adjacent normal tissue (AUC 0.64, 0.52–0.76 versus AUC 0.73, 0.61–0.85).

**Conclusions:**

The DNA methylation profile for e-cigarette use is largely distinct from that of cigarette smoking, did not replicate in independent samples, and was unable to discriminate lung cancer from normal tissue. The extent to which methylation related to long-term e-cigarette use translates into chronic effects requires further investigation.

**Supplementary Information:**

The online version contains supplementary material available at 10.1186/s13148-021-01174-7.

## Introduction

Electronic cigarettes (e-cigarettes) have the potential to reduce smoking-related harm. Although little evidence currently exists on long-term effects, their lack of tar and very low levels of other dangerous substances [1] suggest they are considerably less harmful than cigarettes [2]. They have been shown to be an efficacious [3] and cost-effective [4] smoking cessation aid. While it will take years to fully estimate the impact of e-cigarette use on diseases including cancer, we can investigate whether it is associated with any biomarkers that may predict future health risk [5]. Recent studies have found a reduction in harmful biomarkers among e-cigarette users (vapers) compared with smokers, with some biomarkers showing levels similar to non-smokers [5–7]. However, only a few biomarkers have been investigated and all with relatively short half-lives [8, 9], meaning their utility for predicting long-term effects of e-cigarettes may be limited.

DNA methylation is a type of epigenetic modification involving the addition of methyl groups to the DNA which influences how the underlying sequence is interpreted and expressed. Pronounced differences in methylation have been found between cigarette smokers and non-smokers [10]. These have been replicated in different populations [11, 12] and tissues [13], shown to persist for several years post-cessation [10], are able distinguish tumour from normal samples [14], and are predictive of disease and mortality [15, 16]. Assessing the methylation profile of vaping could therefore inform our understanding of the potential biological impact of their use and the relative health risks compared to cigarettes [17].

In this study, we explored whether e-cigarette use is associated with methylation in saliva and evaluated the degree of similarity between methylation profiles in vapers and cigarette smokers (compared with non-smokers). The investigation of methylation in saliva is supported by the overlap in methylation signals related to smoke exposure in blood and saliva [18], with one study demonstrating a stronger signal in buccal samples compared with matched blood samples [14]. We investigated associations between e-cigarette use and previously-developed methylation scores used to predict smoking-related disease and mortality [12, 16], and biological ageing [19, 20]. We generated a novel methylation score for predicting e-cigarette use and assessed replication in an independent study. We also investigated whether the e-cigarette score was able to distinguish lung tumour and adjacent normal tissue to the same extent as a smoking score, in order to make inferences about the potential importance of e-cigarette-related methylation in lung cancer development.

## Methods

The analysis plan was pre-registered [21] and is summarized in Additional file 1: Figure 1.

### Study Design

The SEE-Cigs study (Studying the Epigenetics of E-cigarette Use) recruited vapers, smokers and non-smokers from the United Kingdom general population. It was important that vapers did not have a long previous smoking history, given the persistence of methylation marks associated with smoke exposure many years after cessation [10, 22]. Vapers were therefore defined as having used e-cigarettes at least weekly for the past 6 months and having smoked < 100 times in their lifetime; smokers as having smoked at least weekly for the past 6 months and having used an e-cigarette < 100 times in their lifetime; and never smokers as having smoked and/or used an e-cigarette < 100 times in their lifetime. We aimed to recruit 120 participants per group (vapers, smokers, non-smokers) to provide > 80% power to detect a 4.5% mean difference in methylation at *p* < 0.05 and > 80% power to detect an 11% mean difference at *p* < 1 × 10^–6^.

### Eligibility criteria

In order to maximise the chance that vapers had never been cigarette smokers, and to minimize confounding by age, we restricted eligibility to between 16 and 35 years old. Additional inclusion criteria were that the participants were in good physical and mental health (measured via self-report) and were able to give informed consent as judged by the investigator. Exclusion criteria obtained via self-report were: dependence on alcohol or drugs (other than nicotine); significant current or past illness (including cancer and type 1/type 2 diabetes); current pregnancy or breast feeding; having a related individual in the sample [21].

### Recruitment

Participants were recruited via a number of mechanisms including from the student population at the University of Bristol, podcasts, blogs, posters/flyers in vape shops, and social media. Recruitment began in January 2017 and was completed in January 2019. The study protocol was originally published on the Open Science Framework on 19/01/2017. On 06/02/2018 we were granted ethics approval to relax the eligibility criteria related to age (16–35 years), and previous smoking history of the vapers and never-smokers (< 100 cigarettes in their lifetime) and vaping history of the smokers and never-smokers (vaped < 100 times in their lifetime). We stated in our original protocol that we would relax age criteria if recruitment stalled. Ethics approval for the study was granted by the Faculty of Science Human Research Ethics Committee at the University of Bristol.

### Consent

All participants have provided written informed consent.

### Questionnaire

Participants answered questions about their smoking and vaping behavior, as well as socio-demographic and behavioural factors including age, gender, height and weight (from which body mass index (BMI) was calculated), ethnicity, educational attainment, occupation and household smoking. They were asked whether they currently used recreational drugs and if so, which recreational drugs they used (stimulants, cocaine, opiates, hallucinogens, cannabis, MDMA/ecstasy). Based on initial responses to the questionnaire, and in accordance with the eligibility criteria, participants were allocated to three participant groups (smokers, vapers and never-smokers). Participants in the ‘smokers’ category were asked whether they smoke cigarettes or roll-ups, whether they were daily or weekly smokers, and how many cigarettes they smoked per day/week as appropriate. They were asked at what time of day they smoke their first cigarette, for how long they had been a smoker, and details about whether they had plans to give up, or whether they had previously attempted to stop smoking. Participants in the ‘vapers’ category were asked about the type of device they used, the nicotine concentration they used most frequently, and whether they had changed the nicotine concentration in the past. If they reported using a refillable device, they were asked to estimate the volume of liquid used in an average day. Similar to the ‘smokers’ group, this group were asked the time of day that they first vape, how long they had been a vaper, and details about whether they had plans to give up vaping. Participants in the ‘never-smokers’ group were asked whether they had ever smoked or vaped, and how frequently, to ensure they met our inclusion/exclusion criteria.

### Sample collection

After completing an online questionnaire, participants were screened for eligibility and sent an information sheet and consent form. On enrolling, participants were posted a study pack containing a saliva collection kit (DNA Genotek Oragene™) from which DNA was extracted and methylation was measured. We supplemented existing kit instructions with a simplified version to aid understanding and improve sample quality, which was posted to participants along with the kit, consent form and information sheet. We asked participants to provide 2 mL of saliva and return the kits through the post to the University of Bristol, where they were processed by the Bristol Bioresource Laboratories.

### DNA methylation profiling

DNA was extracted from the saliva samples and underwent bisulphite conversion using the Zymo EZ DNA Methylation™ kit (Zymo, Irvine, CA). Genome-wide methylation status of over 850,000 cytosine-phosphate-guanine sites (CpGs) was measured using the Illumina HumanMethylationEPIC array according to standard protocol. DNA samples were loaded onto the Illumina HumanMethylationEPIC array in three batches with sampling criteria in place to ensure that all three groups were represented in each batch in order to minimise potential confounding by batch effects. In addition, during the data generation process a wide range of batch variables were recorded in a purpose-built laboratory information management system (LIMS), which also reported quality control (QC) metrics. Microarray data underwent quality control and normalization using *meffil*, an R package designed for pre-processing of large samples of Illumina Methylation BeadChip microarrays [23]. Sample outliers were identified and removed based on sex-chromosome methylation, methylation versus unmethylation intensity, control probes, detection *p* values (N = 10 exclusions in total: 4 vapers, 3 smokers and 3 non-smokers). Poor quality CpG sites, SNP/control probes and CpGs on the sex chromosomes were excluded, resulting in 846,244 CpG sites for analysis.

### Estimated cell type proportions

A cell type reference for saliva was derived as part of *meffil* by combining a white blood cell type reference (GEO: GSE35069) and a buccal cell type reference (GEO: GSE48472). Estimated cell type proportions comprised: Buccal, CD4T, CD8T, Monocytes, B-cells, NK cells and Granulocytes.

### Statistical analysis

#### Epigenome-wide association study (EWAS)

Multivariable linear regression was used to assess the differences in methylation at each measured CpG between (1) vapers versus non-smokers, (2) smokers versus non-smokers, and (3) smokers versus vapers, with adjustment for age, biological sex, BMI, educational attainment, household smoking, recreational drug use and 20 surrogate variables, using *meffil* [23]. We investigated CpGs which reached a Bonferroni-significance threshold of *p* < 5.91 × 10^–8^ (0.05/846,244 CpGs tested), as well as a less stringent threshold of *p* < 1 × 10^–5^. From these EWAS results, we identified differentially methylated regions (DMRs) using the *dmrff* R package [24]. DMRs were defined as regions containing at least two CpGs within 500 bp, each with EWAS meta-analysis *p* values < 0.05 and methylation changes in a consistent direction, and where the regional *p* value surpassed Bonferroni correction.

For the EWAS of vapers versus non-smokers, five additional models were run: (i) with adjustment for estimated cell type composition, (ii) with adjustment for self-reported smoking history (number of cigarettes), (iii) with adjustment for methylation at *AHRR* (cg05575921), an objective biomarker of smoke exposure [25], (iv) after excluding participants with < 60% salivary methylation at *AHRR* (cg05575921), indicative of a substantial smoking history [18], v) restricted to individuals of white ethnicity.

For the CpG sites identified in the EWAS of vapers versus non-smokers, and smokers versus non-smokers, we investigated whether there was evidence of a dose response in methylation levels based on the length of exposure history (6 months–1 year vs > 1 year for e-cigarette use, 6 months–5 years vs > 5 years for smoking).

#### Enrichment and annotation

From the EWAS results of (1) vapers versus non-smokers, and (2) smokers versus non-smokers, we investigated evidence for enrichment of associations among 2,623 and 1,501 smoking-related methylation sites identified in previous large-scale studies of blood [10] and buccal samples [14], using a Wilcoxon rank sum test.

Although we excluded potential participants who reported drug dependence, given the widespread use of e-cigarettes for inhaling cannabinoids [26] and known impact of cannabis on DNA methylation levels [27], we assessed whether any of the 15 CpGs identified in an EWAS of cannabis [27] were associated with e-cigarette use after Bonferroni correction. We also ran an additional model for the EWAS of vapers versus non-smokers with adjustment for reported cannabis use. Further, we assessed whether any CpG sites associated with alcohol use in a previous EWAS [28] were associated with e-cigarette use after Bonferroni correction.

We also investigated whether there was any evidence for replication of 14 CpGs related to e-cigarettes in a previous EWAS [29], and assessed the extent to which the CpGs identified in our EWAS had been previously reported in relation to other traits in two publicly available repositories [30, 31]. We explored the potential functions of the top 50 CpGs identified in each EWAS via GO and KEGG enrichment analysis using the *missMethyl* R package [32].

#### Methylation scores for smoking and epigenetic ageing

Methylation scores can be derived by summing methylation values at relevant CpGs previously identified in relation to a relevant exposure, weighted by the effect sizes observed in independent EWAS studies. Five methylation scores of smoke exposure [10, 14, 16, 25, 33] and four methylation scores of epigenetic ageing [33–36] were quantified.

We assessed associations between scores comprising methylation values derived from a weighted average of CpG sites found to be related to smoking in previous studies. This included scores derived from the CpG sites identified in EWAS conducted by Joehanes et al. [10] and Teschendorff et al. [14], as well as 233 and 172 CpG sites identified in McCartney et al. [16] and Lu et al. [33] respectively. The latter two studies used penalised regression models of smoking pack-years to identify CpG sites most predictive of smoke exposure. Finally, since the CpG site, cg05575921 (*AHRR*) contributed most weight to all of the methylation scores and has been proposed as an independent biomarker of smoking [25], we investigated this site as an additional biomarker. With the exception of *AHRR*, the other scores developed were linear combinations of methylation levels at the relevant CpG sites weighted by the effect sizes of sites identified in relation to smoking from the various studies [10, 14, 16, 33].

For epigenetic ageing, we assessed associations between two “first generation” epigenetic clocks derived from DNA methylation levels at CpG sites found to be strongly associated with chronological age [34, 35], as well as two more recently derived clocks: one optimised to predict physiological dysregulation (PhenoAge) [36] and one optimised to predict lifespan (GrimAge) [33]. To generate the epigenetic ageing measures in SEE-Cigs, we uploaded DNA methylation data for a subset of CpG sites from the Illumina EPIC array to the online DNA Methylation Age Calculator (https://dnamage.genetics.ucla.edu/) developed by the Horvath lab. We also uploaded an annotation file, containing data on chronological age, sex and tissue type (saliva) for the samples. We were able to generate the following epigenetic ageing measures: intrinsic epigenetic age acceleration based on Horvath’s multi-tissue predictor (IEAA) [34]; extrinsic epigenetic age acceleration (EEAA) based on Hannum’s method, which up-weights the contribution of blood cell composition [37]; PhenoAge [36] and GrimAge [33]. Intrinsic epigenetic age acceleration (IEAA) is independent of changes in blood cell composition while extrinsic epigenetic age acceleration (EEAA) incorporates age-related changes in blood cell composition. PhenoAge and GrimAge can be considered as measures of extrinsic ageing.

Multivariable linear regression was used to assess differences in methylation scores between the three groups with adjustment for age, sex, BMI, educational attainment, household smoking and recreational drug use. Further analyses were restricted to individuals of white ethnicity only, with adjustment for methylation-derived smoking pack-years in the GrimAge model [33], and with adjustment for self-reported smoking history when evaluating methylation scores in relation to e-cigarettes.

#### Methylation score for e-cigarette use

We generated methylation scores for e-cigarette use and smoking within SEE-Cigs and then assessed their discriminative performance for predicting e-cigarette use and smoking within SEE-Cigs and in an independent dataset, the Avon Longitudinal Study of Parents and Children (ALSPAC) [38–40]. We also investigated whether the methylation scores for e-cigarette use was able to discriminate tumour from normal tissue in lung to the same extent as the methylation score for smoking, using data from publicly available methylation data in The Cancer Genome Atlas (TCGA) [41].

#### SEE-Cigs

For internal validation of the methylation score of e-cigarette use, we used a training (2/3 sample of vapers and non-smokers) and testing set (1/3 sample of vapers and non-smokers) within the SEE-Cigs study. In the training set, we used the *glmnet* package in R to fit a generalized logistic regression via penalized maximum likelihood using three-fold cross validation and run 10 times to determine a lambda with minimum average error. A methylation score was then generated based on the fitted object produced. The resulting methylation score comprised a sum of the beta-values of the included CpG sites. Its performance in predicting e-cigarette use was evaluated in the test set by generating a receiver operator characteristic (ROC) curve and evaluating the area under the curve (AUC) derived from the logistic regression model using the R package *pROC* (version 1.16.1)*.* We compared the AUC obtained from this model with that from a similar model for predicting smoking. For this, we derived a methylation score for smoking using a training set (2/3 sample of smokers and non-smokers) and testing set (1/3 sample of smokers and non-smokers) within SEE-Cigs.

#### Avon Longitudinal Study of Parents And Children

We next assessed external validation of methylation scores for e-cigarette use and smoking in the Avon Longitudinal Study of Parents and Children (ALSPAC). ALSPAC is a large, prospective cohort study based in the south-west of England. Pregnant women resident in Avon, UK with expected dates of delivery 1st April 1991 to 31st December 1992 were recruited and detailed information has been collected on these women and their offspring at regular intervals [38, 39]. Additional offspring that were eligible to enroll in the study have been subsequently recruited at the ages of 7 and 18 years [40]. The additional enrolment provides a baseline sample of 14,901 offspring who were alive at 1 year of age. Please note that the study website contains details of all the data that are available through a fully searchable data dictionary and variable search tool (http://www.bristol.ac.uk/alspac/researchers/our-data). Ethical approval for the study was obtained from the ALSPAC Ethics and Law Committee and the Local Research Ethics Committees. Consent for biological samples has been collected in accordance with the Human Tissue Act (2004). Informed consent for the use of data collected via questionnaires and clinics was obtained from participants following the recommendations of the ALSPAC Ethics and Law Committee at the time.

When the offspring were 24 years old, they were invited to attend the Focus @ 24 + clinic, which took place between June 2015 and October 2017. 4,026 were seen at this clinic, where fasting blood samples were taken. Blood samples from 570 of the offspring were selected for DNA methylation profiling to maximize overlap with existing DNA methylation profiles generated collected at younger ages as part of the Accessible Resource for Integrated Epigenomic Studies (ARIES) [42]. Following DNA extraction, samples were bisulphite-converted using the Zymo EZ DNA MethylationTM kit (Zymo, Irvine, CA). Genome-wide methylation was then measured using Illumina Infinium MethylationEPIC Beadchip arrays. The arrays were scanned using an Illumina iScan, with initial quality review using GenomeStudio. During the data generation process a wide range of batch variables were recorded in a LIMS, which also reported quality control metrics. Quality control and normalization was then carried out using the *meffil* R package [23]. Quality control included checks for sample swaps using genotype matching and sex prediction, methylated versus unmethylated signal outliers, dye bias, poor probe signal detection, and low bead numbers. Only one sample failed and was excluded due to evidence of being a sample swap and having multiple control probe outliers. The 569 samples that passed were normalized in *meffil* using functional normalization using the top 20 control probe principal components and sample plate as a random effect.

At the same time point when the offspring were 24 years old, information on smoking and e-cigarette use was obtained from a questionnaire that was completed by 458 of the offspring with DNA methylation data. Questionnaire data were collected and managed using REDCap electronic data capture tools hosted at the University of Bristol [43]. The participants were asked whether they had ever smoked or used an e-cigarette, as well as about the frequency and duration of use. From these details were determined three groups of participants: (1) vapers (currently users of electronic cigarettes or other vaping devices, *n* = 14) (2) smokers (current daily or weekly smokers, *n* = 47) (3) non-smokers (smoked < 100 cigarettes in their lifetime and never used an electronic cigarette or other vaping device, *n* = 262).

We assessed the discriminative performance of a methylation score generated in SEE-Cigs for predicting e-cigarette use (vs. non-smoking) in the ALSPAC cohort, which was compared with a methylation score for predicting smoking (vs. non-smoking). Both e-cigarette and smoking methylation scores were obtained using the same approach described above in the full sample of: i) vapers vs. non-smokers and ii) smokers versus non-smokers, respectively, in SEE-Cigs.

#### The Cancer Genome Atlas

We used data on 27 individuals with lung adenocarcinoma (LUAD) and 41 individuals with lung squamous cell carcinoma (LUSC) who had Illumina Infinium 450K DNA methylation measured in both tumour and adjacent normal samples as part of The Cancer Genome Atlas (TCGA). Again, a methylation score was generated on the full sample of vapers versus non-smokers in SEE-Cigs, this time restricted to CpG sites which were present only on the 450K array. This was compared with a methylation score for smoking generated on the full sample of smokers versus non-smokers, again restricted to 450K CpG sites.

## Results

### Descriptive characteristics

**Fig. 1:**
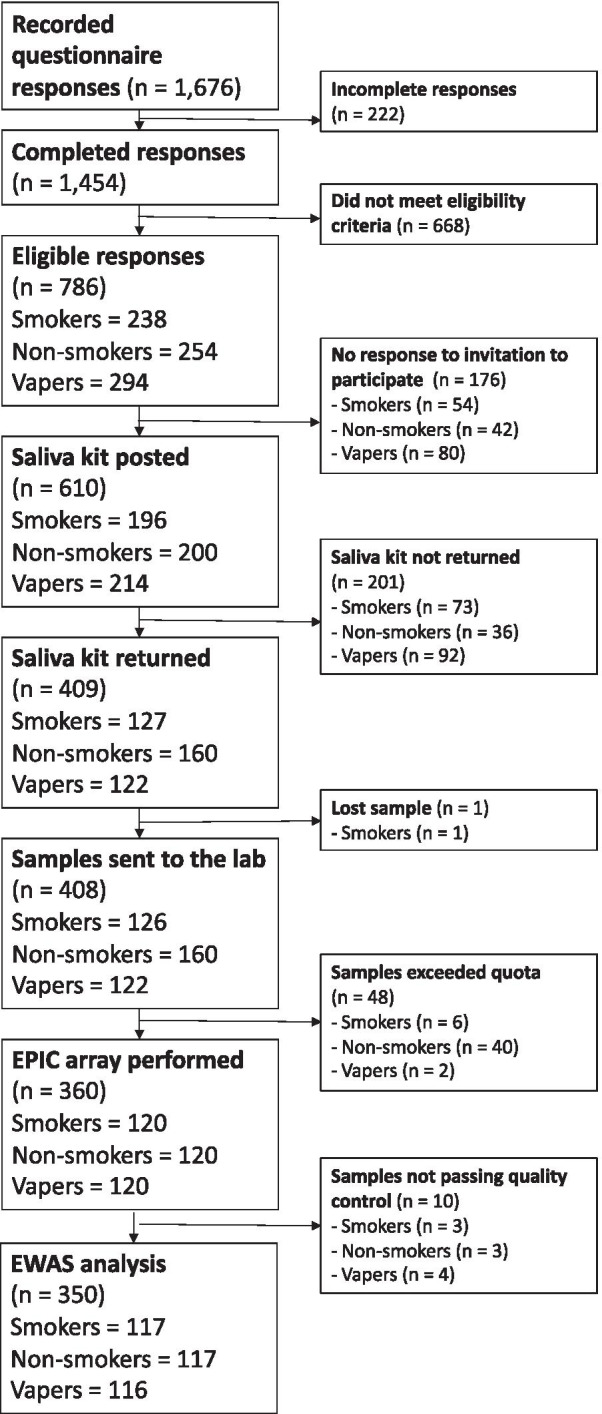
Participant flow chart for SEE-Cigs

**Fig. 2:**
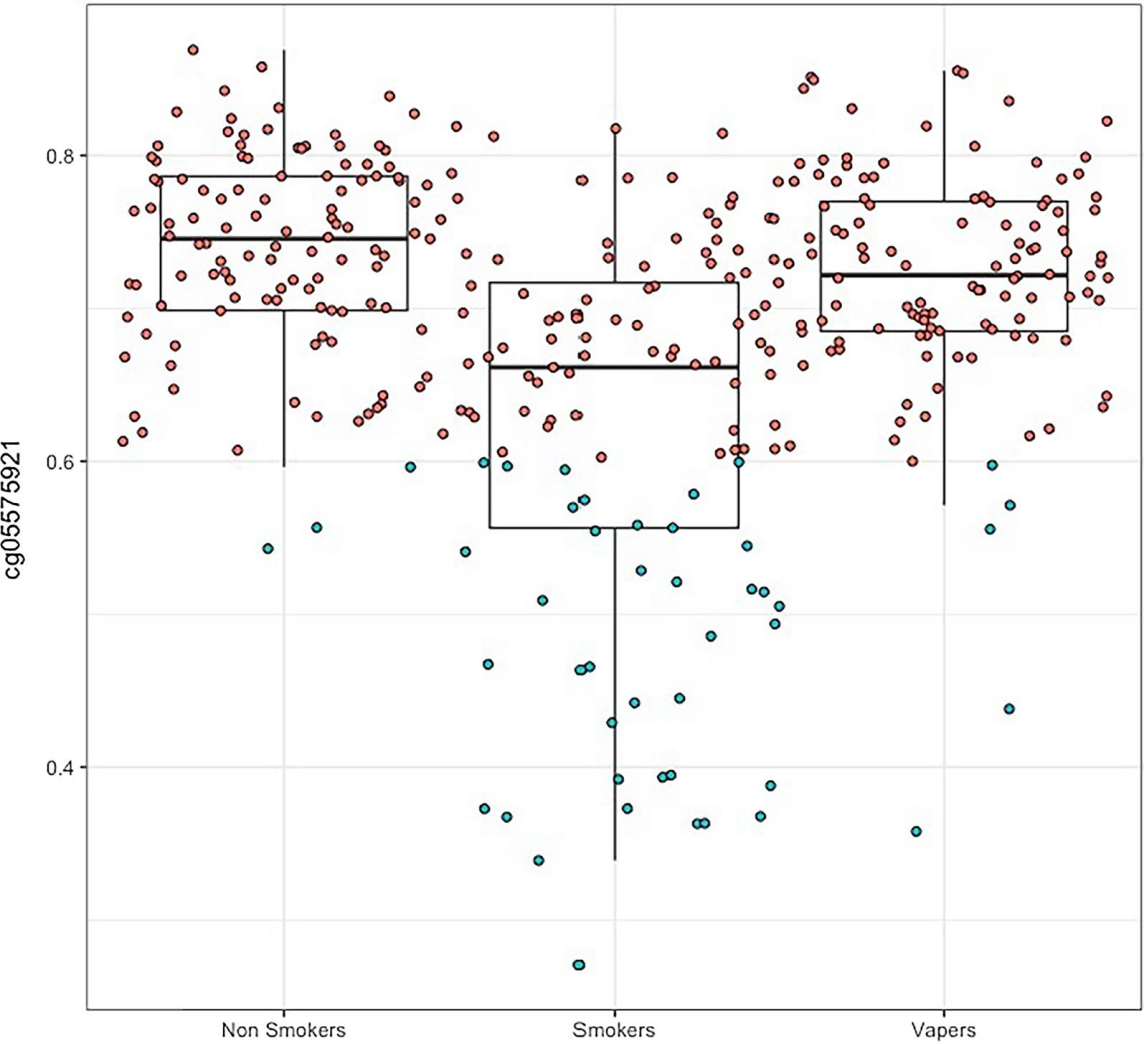
*AHRR* (cg05575921) methylation among participant groups < 60% methylation (shown in blue) is indicative of a substantial smoking history

Figure 1 shows the participant flow for the SEE-Cigs study. The final sample consisted of 117 smokers, 117 non-smokers and 116 vapers with methylation data. Descriptive characteristics are displayed in Table 1. Compared with non-smokers, vapers were more likely to have higher BMI, be male, have lower educational attainment and be more exposed to household smoke. Smokers were more likely to be male, have lower educational attainment, be more exposed to household smoke and to use drugs recreationally. Smokers were slightly older on average than non-smokers and vapers. The majority of participants were of white ethnicity, with a slightly higher proportion of non-white individuals among the non-smokers. Smokers had smoked for a median of 1.20 (IQR = 0.38–3.15) pack-years, while both non-smokers and vapers reported a minimal smoking history. This was verified based on levels of *AHRR* methylation (cg05575921), for which 3 non-smokers and 5 vapers had < 60% salivary methylation, indicative of previous smoking (Fig. 2). There were no differences in cell type proportions of the saliva samples obtained from the participants. Most vapers used e-cigarettes containing nicotine and vaped daily.

**Table 1:** Descriptive characteristics of participant groups in this study

	Non-smokers (*n* = 117^a^)	Smokers (*n* = 117^a^)	Vapers (*n* = 116^a^)
	Mean (SD)	Mean (SD)	Mean (SD)
Socio-demographic factors			
Age (years) (*n* = 348)	20.6 (3.5)	22.8 (4.9)	20.9 (4.3)
BMI (kg/m^2^) (*n* = 345)	22.6 (3.5)	23.2 (4.2)	26.0 (6.8)

	Median (IQR)	Median (IQR)	Median (IQR)
Smoking (pack-years) (*n* = 313)	0 (0, 0.0001)	1.20 (0.38, 3.15)	0.0013 (0.0006, 0.0027)

	*n* (%)	*n* (%)	*n* (%)
Sex (% male) (*n* = 350)	60 (50)	60 (50)	73 (80)
Ethnicity (% white) (*n* = 344)	94 (80.3)	103 (88.8)	99 (89.2)
Education (% higher education) (*n* = 344)	101 (86.3)	93 (80.2)	69 (59.5)
Occupation (% unemployed) (*n* = 344)	5 (4.3)	10 (8.6)	21 (18.9)
Household smoking (% yes) (*n* = 346)	47 (40.2)	79 (67.5)	76 (67.9)
Recreational drug use (% yes) (*n* = 346)	7 (6.0)	31 (27.0)	11 (9.7)
Cannabis (% yes) (*n* = 346)	5 (4.3)	23 (20.0)	10 (8.7)

	Mean (SD)	Mean (SD)	Mean (SD)
Estimated cell type proportions (%) (*n* = 350)			
Buccal	48.3 (18.2)	49.4 (18.5)	45.4 (17.1)
Bcell	1.7 (1.2)	2.1 (1.8)	2.2 (2.1)
CD4T	0.2 (0.4)	0.2 (0.5)	0.2 (0.7)
CD8T	0.001 (0.005)	0.04 (0.44)	0.03 (0.24)
Granulocytes	47.3 (19.8)	45.4 (20.8)	49.1 (19.3)
Mono	0.4 (0.7)	0.5 (0.9)	0.6 (1.0)
NK	1.1 (1.4)	1.5 (2.6)	1.6 (2.3)

	*n* (%)	*n* (%)	*n* (%)
Vaping characteristics			
E-cigarette device (*n* = 113)	N/A	N/A	
Cigalike (1st generation)			2 (1.7)
Tanks (2nd generation)			11 (9.4)
Mods (3rd generation)			76 (67.3)
Pods (4th generation)			17 (15)
Other			7 (6.2)
Frequency of use (% daily) (*n* = 113)			105 (92.9)
Device contains nicotine (% yes) (*n* = 112)			108 (96.4)
Increased nicotine concentration in the past (% yes) (*n* = 113)			16 (14.2)
Length of time vaping (% > 1 year) (*n* = 113)			70 (62.0)

	Mean (SD)	Mean (SD)	Mean (SD)
Nicotine content (mg/ml) (*n* = 112)	N/A	N/A	5.6 (4.7)
Liquid per day (ml) (*n* = 108)			7.8 (7.7)

	*n* (%)	*n* (%)	*n* (%)
Smoking characteristics			
Cigarettes or roll-ups (% roll ups) (*n* = 117)		87 (74.4)	
Frequency of smoking (% daily) (*n* = 117)		99 (84.6)	
Length of time smoking (% > 5 years) (*n* = 114)		52 (45.6)	

^a^Maximum sample sizes

### EWAS

7 CpGs were associated with vaping (vs. non-smoking) at *p* < 1 × 10^–5^ and none at *p* < 5.91 × 10^–8^ (Table 2). The top three CpGs were located in protein-coding genes for a ribonuclease P/MRP subunit (*RPP14*), an insulin-like growth factor receptor (*IGF1R*) and a gamma-aminobutyric acid (GABA) A receptor (*GABRP*). After accounting for cell composition, smoking history, ethnicity and cannabis use, associations at the 7 CpGs weakened slightly, with the exception of signals at cg12435725 (*RPP14*), which strengthened with adjustment for self-reported smoking history (*p* = 9.02 × 10^–8^) and *AHRR* methylation (*p* = 8.92 × 10^–8^); cg02066693, which was stronger among individuals of white ethnicity (*p* = 6.27 × 10^–7^); and cg12734956, which was stronger after excluding individuals with low levels of *AHRR* methylation (*p* = 3.27 × 10^–7^) (Additional file 2: Table 1). 13 CpGs were associated with smoking (vs. non-smoking) at *p* < 1 × 10^–5^ and one at *p* < 5.91 × 10^–8^: cg05575921 located in *AHRR* encoding the aryl hydrocarbon receptor repressor (Additional file 2: Table 2). 14 CpGs were associated with vaping (vs. smoking) at *p* < 1 × 10^–5^, with the top association also at *AHRR* (cg05575921) (Additional file 2: Table 3). Methylation at cg05575921 was 8.2% (95% CI = 5.7–10.5) lower in smokers compared with non-smokers, and 7.1% (95% CI = 4.6–9.6) lower in smokers compared with vapers. 31 DMRs were identified in vapers versus non-smokers, 39 in smokers versus non-smokers and 81 in vapers versus smokers. The top DMR identified between vapers and non-smokers comprised 9 CpGs in *MUC4*, with higher methylation in vapers compared with non-smokers (7.3% (95% CI = 5.7–9.0; *p* = 4.13 × 10^–18^) (Additional file 2: Table 4).

**Table 2:** Differentially methylated CpG sites associated with e-cigarette use versus non-smoking

CpG site	Chromosome	Position	Gene Symbol	Beta	SE	*p* value*
cg12435725	3	58293450	*RPP14*	− 0.060	0.012	6.43 × 10^–7^
cg02066693	15	99366135	*IGF1R*	− 0.019	0.004	5.47 × 10^–6^
cg14872828	5	170210761	*GABRP*	− 0.035	0.007	2.77 × 10^–6^
cg12734956	12	112181430	*ACAD10*	− 0.013	0.003	5.89 × 10^–6^
cg02934082	3	122705793	*SEMA5B*	− 0.036	0.008	5.92 × 10^–6^
cg00388391	1	18459578	*IGSF21*	0.025	0.006	8.38 × 10^–6^
cg10440286	22	37771664	*ELFN2*	0.044	0.010	9.31 × 10^–6^

Model adjusted for age, sex, body mass index, educational attainment, household smoking, recreational drug use and 20 surrogate variables. *n* = 111 vapers, *n* = 117 non-smokers

*p values < 1 × 10^–5^

**Fig. 3:**
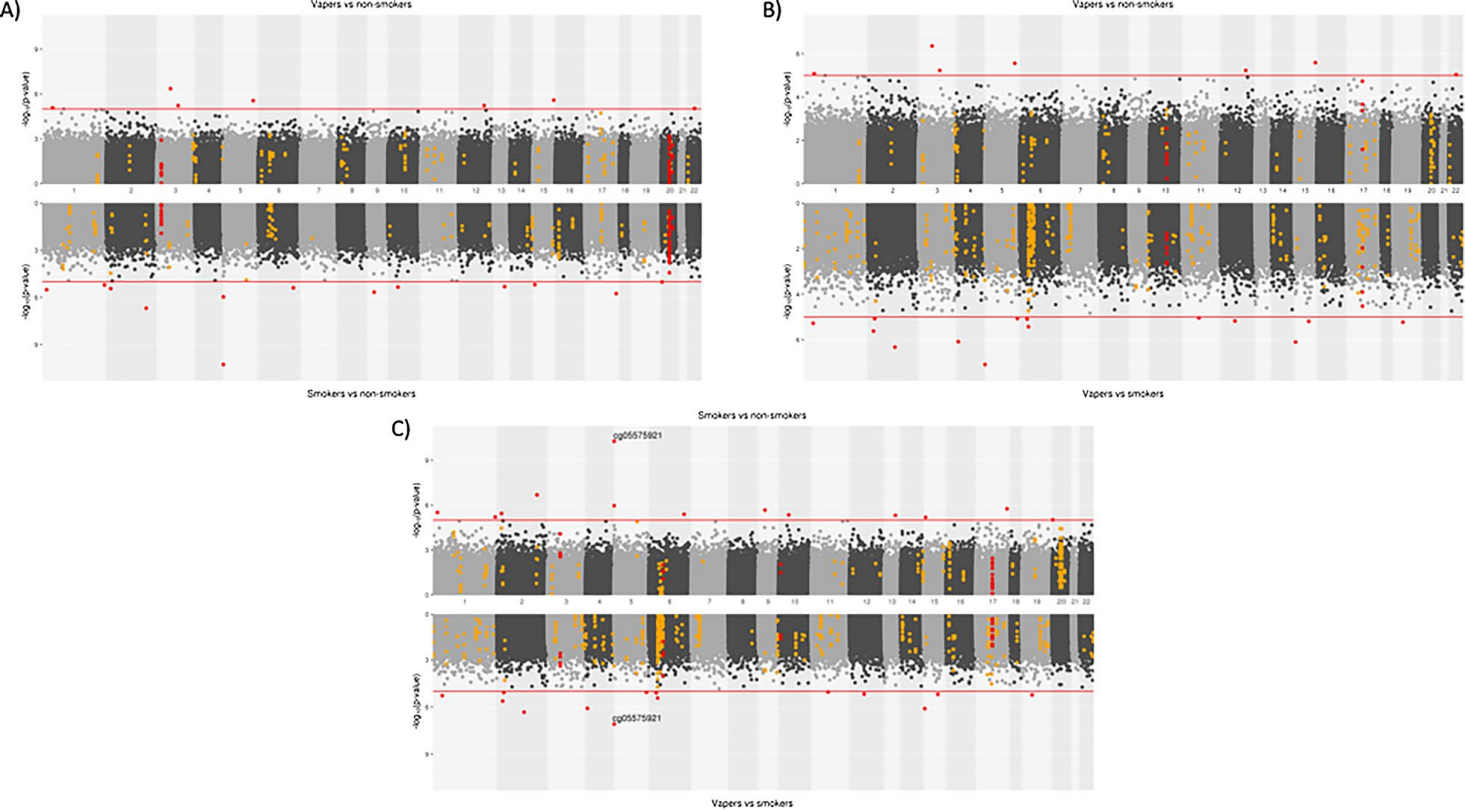
Comparison of epigenome-wide associations studies. **A** EWAS for e-cigarette use (vs. non-smoking) and smoking (vs. non-smoking). **B** EWAS for e-cigarette use (vs. non-smoking) and e-cigarette use (vs. smoking). **C** EWAS for smoking (vs. non-smoking) and e-cigarette use (vs. smoking)

Apart from associations at *AHRR* in the models involving smoking, there was limited overlap in the top CpGs identified in the three EWAS (Fig. 3, Additional file 1: Figure 2). 9 DMRs were found in common between at least two of the EWAS models (Fig. 3). One DMR was hypermethylated (chr20:*BLCAP;NNAT*) and two hypomethylated (chr20:*SLC2A10* and chr3:*THRB*) in non-smokers compared with vapers and smokers. Two DMRs were hypermethylated (chr10 and chr3:*CACNA1D*) and two were hypomethylated (chr17:*BRCA1;NBR2* and chr6:*PRRT1;PPT2*) in smokers compared with non-smokers and vapers. Two DMRs were hypermethylated in vapers compared with smokers and non-smokers (chr10:*ANXA11;LINC00857* and chr17:*HSPB9;KAT2A*).

For the 7 CpG sites associated with vaping (vs. non-smoking), the direction of effect was consistent irrespective of vaping duration. The magnitude of effect was typically larger among those participants reporting to have vaped for > 1 year compared with those vaping for 6 months–1 year, with the exception of cg10440286 (*ELFN2*). For the 13 CpG sites associated with smoking (vs. non-smoking), the direction of effect was consistent irrespective of smoking duration. The magnitude of effect was larger among those participants reporting to have smoked for > 5 years compared with those smoking for 6 months–5 years at some (e.g. cg05575921 (*AHRR*), cg21732535 (*PTH2R*)), but not all (e.g. cg23771956, cg13159505 (*RPTOR*)) sites (Additional file 1: Figure 3).

### Enrichment and annotation

There was strong enrichment of known smoking-related CpGs in the EWAS of smokers versus non-smokers (Wilcoxon test *p* = 1.05 × 10^–14^ for Joehanes et al. CpGs [10] and *p* = 1.10 × 10^–9^ for Teschendorff et al. CpGs [14]) (Additional file 1: Figure 4), unlike in the EWAS of vapers versus non-smokers (Joehanes *p* = 0.67, Teschendorff *p* = 0.28) (Additional file 1: Figure 4). One smoking-related CpG was found to be similarly methylated in vapers in *GABRP* (cg14872828; *p* = 2.77 × 10^–6^).

One CpG previously associated with cannabis was found to be associated with e-cigarettes after Bonferroni correction (cg04180046; *p* = 0.0029). This CpG has also been previously associated with smoke exposure [10], and there was no difference in methylation at this site between vapers and smokers in the present study (*p* = 0.865) (Additional file 2: Table 5). No CpGs previously associated with alcohol use were found to be associated with e-cigarettes after Bonferroni correction (*p* > 0.005) (Additional file 2: Table 6). We also found little evidence of associations between 14 CpGs previously found in relation to vaping [29] in any of the EWAS after Bonferroni correction (*p* > 0.01) (Additional file 2: Table 7).

Three of the seven CpGs associated with e-cigarettes have been identified in previous EWAS for smoking, Down Syndrome, systemic corticosteroid, prostate cancer, gestational age and fetal versus adult liver (Additional file 2: Tables 8 and 9). We found limited enrichment for KEGG pathways or GO terms (false discovery rate, FDR *p* > 0.05) (Additional file 2: Tables 10–13). In relation to e-cigarette use, response to ethanol/alcohol, positive regulation of insulin secretion and GABA transport were the top GO terms. Butanoate metabolism, synaptic vesicle cycle and GABAergic synapse were the top KEGG pathways.

### Methylation scores for smoking

All of the methylation scores for smoking were correlated with reported pack-years smoked (|*r*|= 0.21–0.52, *p* < 0.0001), except the Teschendorff score [14] (*r* = 0.07, *p* = 0.19) (Additional file 2: Table 14). All of the scores differed between the smokers versus non-smokers (0.40–0.90 SD), and vapers versus smokers (0.37–0.86 SD). While neither the Joehanes [10] nor the Teschendorff score [14] differed between vapers versus non-smokers, higher levels of the other three scores (Lu [33], McCartney [16], *AHRR*) were observed in vapers versus non-smokers (0.21–0.30 SD) (Table 3). However, these associations attenuated when reported smoking history was included in the model (Additional file 2: Table 15). Associations were similar in analyses restricted to individuals of white ethnicity (Additional file 2: Table 16).

**Table 3:** Differences in DNA methylation scores between participant groups

DNA methylation score	Smokers versus non-smokers (*n* = 231)		Vapers versus non-smokers (*n* = 228)		Vapers versus smokers (*n* = 225)	
	Coefficient (SE)	*p* value	Coefficient (SE)	*p* value	Coefficient (SE)	*p* value
Smoking^a^						
Joehanes [10]	0.62 (0.15)	4.39 × 10^–5^	0.13 (0.15)	0.360	− 0.59 (0.14)	3.25 × 10^–5^
Teschendorff [14]	0.40 (0.16)	0.010	0.12 (0.14)	0.864	− 0.34 (0.15)	0.024
Lu [33]	0.65 (0.11)	7.9 × 10^–9^	0.30 (0.10)	0.002	− 0.47 (0.13)	1.98 × 10^–4^
McCartney [16]	0.83 (0.13)	2.14 × 10^–10^	0.30 (0.12)	0.012	− 0.68 (0.13)	3.67 × 10^–7^
*AHRR* [25]	− 0.90 (0.13)	1.12 × 10^–11^	− 0.23 (0.11)	0.028	0.82 (0.14)	1.62 × 10^–9^
Epigenetic age^b^						
IEAA [34]	− 0.02 (0.68)	0.981	− 0.08 (0.64)	0.907	0.41 (0.67)	0.538
EEAA [37]	0.91 (0.73)	0.212	0.38 (0.69)	0.578	− 0.68 (0.69)	0.325
PhenoAge [36]	0.26 (0.81)	0.751	− 0.20 (0.86)	0.816	0.11 (0.85)	0.897
GrimAge [33]	2.57 (0.59)	1.36 × 10^–5^	0.74 (0.55)	0.179	− 2.41 (0.57)	2.09 × 10^–5^

Adjusted for age, sex, body mass index, educational attainment, household smoking and recreational drug use

^a^Coefficients = SD unit difference in score between groups; ^b^coefficients = difference in years between groups

### Methylation scores for epigenetic ageing

Epigenetic age estimates were strongly correlated with chronological age (*r* = 0.49–0.70, *p* < 0.0001) (Additional file 2: Table 17). There was little difference in estimates of epigenetic age acceleration (EAA) between the three groups, except for GrimAge, where smokers had higher EAA relative to both vapers (2.5, 95% CI = 1.4–3.6 years) and non-smokers (2.6, 95% CI = 1.4–3.7 years) (Table 3). Associations persisted in analyses adjusting for a methylation score for smoking pack-years [33] (Additional file 2: Table 18) and when restricted to individuals of white ethnicity (Additional file 2: Table 16).

### Methylation scores for e-cigarette use

We generated methylation scores for predicting e-cigarette use (Additional file 2: Table 19). These performed poorly at discriminating vapers from non-smokers in both the internal (AUC = 0.55, 95% CI = 0.41–0.69) and external validation sets (AUC = 0.57, 95% CI = 0.36–0.74). This was in contrast to the high discriminative performance of the smoking scores (internal AUC = 0.80, 95% CI = 0.69–0.91 and external AUC = 0.80, 95% CI = 0.72–0.88; Additional file 1: Figure 5). The smoking and e-cigarette methylation scores performed poorly at discriminating tumour from adjacent normal tissue in lung adenocarcinoma (LUAD) cases (AUC = 0.57, 95% CI = 0.40–0.73 and 0.58, 95% CI = 0.42–0.74). The e-cigarette methylation score also performed poorly at discriminating tumour from adjacent normal tissue in lung squamous cell carcinoma (LUSC) cases (0.64, 95% CI = 0.52–0.76). Moderate discrimination was observed from the smoking methylation score in LUSC cases (0.73, 95% CI = 0.61–0.85) (Additional file 1: Figure 6).

## Discussion

Among 117 smokers, 117 non-smokers and 116 vapers with a limited smoking history, we found that salivary methylation signals of e-cigarette use were weak and largely distinct from those established in relation to cigarette smoking. The top 3 CpGs for vaping were located in protein-coding genes for a ribonuclease P/MRP subunit (*RPP14*) (*p* = 6.43 × 10^–7^), an insulin-like growth factor receptor (*IGF1R*) (*p* = 5.47 × 10^–6^) and a gamma-aminobutyric acid (GABA) A receptor (*GABRP*) (*p* = 2.77 × 10^–6^). The top DMR was located in *MUC4* encoding Mucin 4 (*p* = 4.13 × 10^–18^), an integral membrane glycoprotein present in mucus upregulated in vapers [44]. Of the DMRs found to be differentially methylated in vapers compared with both smokers and non-smokers, *ANXA11* suggestively plays an important role in lung function, and variation in this gene has been associated with Sarcoidosis (mainly affecting the lung) [45] and Chronic Obstructive Pulmonary Disease-related biomarkers [46]. Ethanol/alcohol, positive regulation of insulin secretion, GABA transport and butanoate metabolism were among the most enriched pathways, reflecting biological responses to e-cigarette constituents (ethyl alcohol, ethyl butyrate and nicotine). Methylation scores for smoking and biological ageing were similar between vapers and non-smokers. Higher levels of a biological ageing score (GrimAge) were observed in smokers. Finally, a methylation score generated to index e-cigarette use poorly discriminated vapers from non-smokers in SEE-Cigs and in an independent dataset (ALSPAC), which was in contrast to a methylation score generated to index smoking. The smoking methylation score also showed better discrimination of tumour and adjacent normal tissue in lung squamous cell cases compared with the e-cigarette methylation score.

In contrast to our findings, two studies (comprising 32 and 45 participants, respectively) found associations between e-cigarettes and methylation levels which overlap with smoking-related signals [29, 47]. We were also unable to replicate methylation differences for 14 CpGs previously related to e-cigarettes [29]. However, it is important to highlight that the vapers included in the previous studies were not selected for smoking history as stringently as in the current study and former smokers were likely to comprise a substantial proportion of the sample.

Two studies showing a weak methylation profile related to smokeless sources of nicotine are supportive of our results [48, 49]. However, both studies only investigated peripheral blood and not tissue-specific methylation at the site of exposure (e.g. saliva).

Acceleration of a biological ageing methylation score in smokers but not vapers is of interest since such markers are predictive of age-related disease and mortality independent of chronological age [37, 50, 51]. Discrimination of lung tumour and adjacent samples by a salivary-based methylation score for smoking is supported by previous findings [14]. The lack of discrimination by the e-cigarette methylation score could indicate that smoking-related methylation changes may be more relevant to tumourigenesis than changes related to e-cigarettes. However, the smoking methylation score generated in the present study was lower in lung squamous cell carcinoma relative to normal tissue, the inverse of what was expected due to higher levels being observed in smokers [14]. We have previously found methylation in tumour tissue is in the opposite direction to that observed in relation to smoking for *AHRR* (cg05575921) [52] (Additional file 1: Figure 7), the CpG contributing most weight to the methylation score for smoking (Additional file 2: Table 19).

Major strengths relate to the design of the study, including the recruitment of individuals with a limited smoking history and the assessment of methylation levels in an easily accessible and exposure-relevant tissue for investigating epigenetic profiles of e-cigarettes. Limitations include the representativeness of our study sample, with demographic characteristics different to the general population due to the strict inclusion criteria. The young age of the study sample (mean age = 21 years) and limited smoking and vaping history could hamper the detection of methylation signals. This may explain why so few CpGs were identified in relation to smoking than anticipated based on previous studies of oral samples [14]. Nonetheless, enrichment of smoking-related CpGs among the smokers indicates that these signals were present but more weakly associated. Furthermore, small sample size could have hindered the detection and replication of e-cigarette methylation signals, in particular in the external validation analysis in ALSPAC where methylation data was only available on 14 vapers. Similar enrichment for smoking-related CpGs was not found among the vapers, indicating that the methylation signature of e-cigarettes is distinct from that of smoking, and that vapers in the present study were accurate in reporting their limited smoking history, despite this not being biochemically verified.

While it appears that the methylation profile of vapers is less pronounced than that of smokers, the methylation changes associated with e-cigarettes may be commensurate in scale with other lifestyle exposures and replication of the signals identified in relation to e-cigarettes in larger studies is warranted. In addition, future studies may benefit from comparing saliva methylation patterns in e-cigarette users with those from other sample types, such as blood, since some markers may perform better as predictors when measured in whole blood [18].

Additional research in cohort studies is required to investigate methylation changes among ex-smokers quitting with different methods, including e-cigarettes. While findings from this study suggest that e-cigarettes may have distinct health effects from cigarettes, we cannot provide robust conclusions regarding the safety of e-cigarettes. Furthermore, although the methylation changes identified in relation to both smoking and e-cigarettes may be predictive of future disease risk, the causal consequences of these methylation changes on health outcomes are currently uncertain [52].

## Conclusions

Findings from this study suggest that e-cigarette use does not impact saliva methylation in the same way as cigarette smoking. Unlike for smoking, the methylation profile for e-cigarettes did not replicate in independent samples and was not able to discriminate cancer from normal tissue. However, the short duration of e-cigarette use by the study participants and sample size may have hampered the detection of signals. Further studies are required to detect a robust methylation signature for long-term e-cigarette use. The extent to which methylation related to e-cigarette use translates into chronic effects and relevant health outcomes should also be investigated.

## Supplementary Information

**Additional file 1.** Supplementary figures.

**Additional file 2.** Supplementary tables.

## Data Availability

The underlying methylation and phenotypic data used in this study cannot be made publicly available since the data contain information that could compromise participant consent and confidentiality. Full summary data from the epigenome-wide association studies conducted as part of the main analysis have been uploaded to the EWAS Catalog (http://www.ewascatalog.org/).
