# Supplementary material for: Investigating the DNA methylation profile of e-cigarette use
Source: Clin Epigenetics. 2021 Sep 28;13:183. doi: 10.1186/s13148-021-01174-7 (PMC8479883; doi:10.1186/s13148-021-01174-7)
Supplement: Supplementary file 1 — Additional file 1. Supplementary figures. [file 13148_2021_1174_MOESM1_ESM.docx]

**Supplementary Figure 1** – Analysis plan

**Supplementary Figure 2 -** Heatmap of differentially methylated CpG sites identified among smokers, vapers and non-smokers

**Supplementary Figure 3 -** Dose-response assessment of differentially methylated CpG sites between **a)** vapers vs. non-smokers **b)** smokers vs. non-smokers by duration of exposure

**Supplementary Figure 4** – Enrichment of known smoking related CpG sites in SEE-Cigs

A) B)


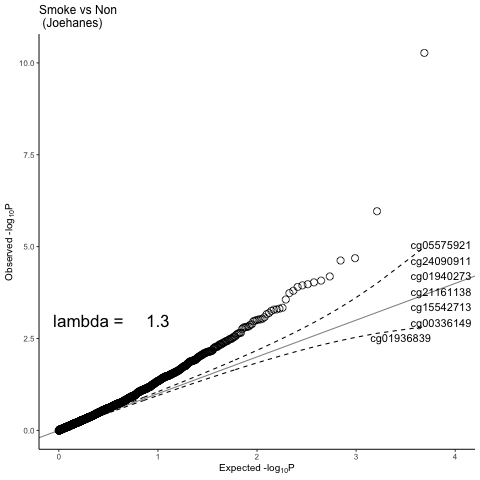

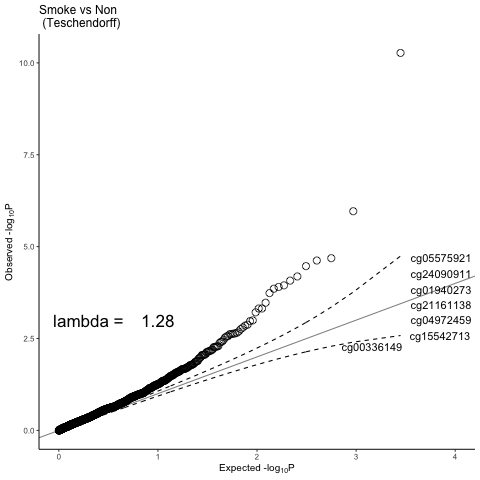


C) D)


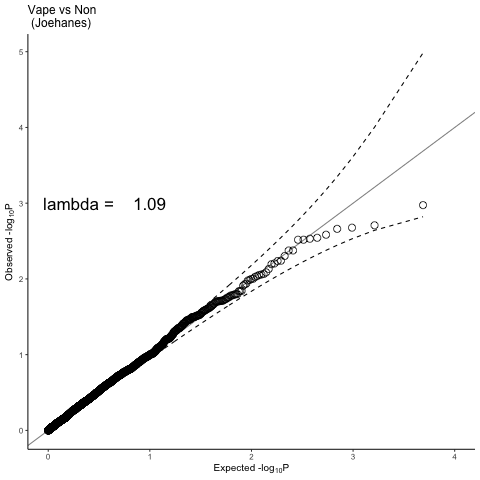

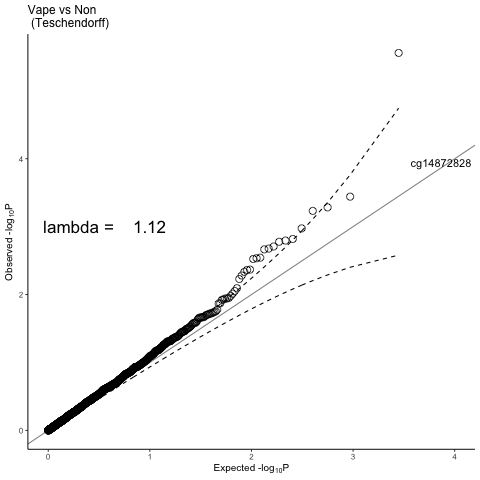


1. CpG sites from Joehanes et al (*1*) in EWAS of smokers vs non-smokers in SEE-Cigs
2. CpG sites from Teschendorff et al (*2*) in EWAS of smokers vs non-smokers in SEE-Cigs
3. CpG sites from Joehanes et al (*1*) in EWAS of vapers vs non-smokers in SEE-Cigs
4. CpG sites from Teschendorff et al (*2*)in EWAS of vapers vs non-smokers in SEE-Cigs

**Supplementary Figure 5 -** Comparing the discriminative performance of a DNAm score for e-cigarette use with a DNAm score for smoking in independent samples

1. Performance of a DNAm score for smoking in discriminating smokers (1) from non-smokers (0) in SEE-Cigs (n= 32 smokers and n= 32 non-smokers)
2. Performance of a DNAm score for e-cigarette use in discriminating vapers (1) from non-smokers (0) in SEE-Cigs (n= 32 vapers and n= 32 non-smokers)
3. Performance of a DNAm score for smoking in discriminating smokers (1) from non-smokers (0) in ALSPAC (n= 47 smokers and n= 262 non-smokers)
4. Performance of a DNAm score for e-cigarette use in discriminating vapers (1) from non-smokers (0) in ALSPAC (n= 14 vapers and n= 262 non-smokers)

**Supplementary Figure 6 -** Comparing the performance of a DNAm score for e-cigarette use with a DNAm score for smoking in discriminating lung tumour from normal adjacent tissue

1. Performance of a DNAm score for smoking in discriminating lung adenocarcinoma (LUAD) tumour (T) from adjacent normal (N) in TCGA (n= 27 matched pairs)
2. Performance of a DNAm score for e-cigarette use in discriminating lung adenocarcinoma (LUAD) tumour (T) from adjacent normal (N) in TCGA (n= 27 matched pairs)
3. Performance of a DNAm score for smoking in discriminating lung squamous cell carcinoma (LUSC) tumour (T) from adjacent normal (N) in TCGA (n= 40 matched pairs)
4. Performance of a DNAm score for e-cigarette use in discriminating lung squamous cell carcinoma (LUSC) tumour (T) from adjacent normal (N) in TCGA (n= 40 matched pairs)

**Supplementary Figure 7** – Assessing the discriminative performance of AHRR (cg05575921) methylation

1. Performance of *AHRR* (cg05575921) methylation in discriminating smokers (1) from non-smokers (0) in SEE-Cigs (n= 32 smokers and n= 32 non-smokers)
2. Performance of *AHRR* (cg05575921) methylation in discriminating smokers (1) from non-smokers (0) in ALSPAC (n= 47 smokers and n= 262 non-smokers)
3. Performance of *AHRR* (cg05575921) methylation in discriminating lung adenocarcinoma (LUAD) tumour (T) from adjacent normal (N) in TCGA (n= 27 matched pairs)
4. Performance of *AHRR* (cg05575921) methylation in discriminating lung squamous cell carcinoma (LUSC) tumour (T) from adjacent normal (N) in TCGA (n= 40 matched pairs)

1. R. Joehanes *et al.*, Epigenetic Signatures of Cigarette Smoking. *Circ Cardiovasc Genet* **9**, 436-447 (2016).

2. A. E. Teschendorff *et al.*, Correlation of Smoking-Associated DNA Methylation Changes in Buccal Cells With DNA Methylation Changes in Epithelial Cancer. *JAMA Oncol* **1**, 476-485 (2015).
